# Maralixibat for the treatment of severe xanthomas in two children with Alagille syndrome: Case reports

**DOI:** 10.1002/jpr3.70105

**Published:** 2025-11-23

**Authors:** Geetanjali Bora, Ruben E. Quiros‐Tejeira, Bernadette Vitola

**Affiliations:** ^1^ Division of Pediatric Gastroenterology Hepatology and Nutrition, Medical College of Wisconsin Milwaukee Wisconsin USA; ^2^ Division of Gastroenterology Hepatology, and Nutrition, Children's National Hospital Washington District of Columbia USA; ^3^ Department of Pediatrics, Children's Nebraska University of Nebraska Medical Center Omaha Nebraska USA

**Keywords:** cholestasis, ileal bile acid transporter inhibitor, pruritus

## Abstract

Alagille syndrome (ALGS) is a rare, autosomal dominant disorder which presents with a broad range of clinical manifestations, including cholestatic pruritus. A unique manifestation of ALGS is the presence of xanthomas in 24%–42% of patients, which can lead to liver transplantation. Maralixibat, an ileal bile acid transporter (IBAT) inhibitor, has demonstrated improvements in both cholestatic pruritus and xanthomas in clinical trials. We report here on the use of maralixibat in two patients with ALGS and unusual manifestations of xanthomatosis, including one patient with airway xanthomas and a second patient with severe, diffuse xanthomas. In both cases, almost complete resolution of severe, debilitating xanthomas and clinically meaningful improvements in pruritus and serum bile acid levels were observed after up to 1 year of treatment with maralixibat. These cases support the utilization of maralixibat for the management of ALGS beyond cholestatic pruritus.

## Abstract (highlights)

**What is Known:**

- Maralixibat is approved by the US Food and Drug Administration for treatment of cholestatic pruritus in patients with Alagille syndrome (ALGS) ≥ 3 months of age in the United States and ≥2 months of age in the European Union.
- In a clinical trial with maralixibat in patients with ALGS, significant improvements in pruritus and xanthomas were observed.

**What is New:**

- These cases demonstrate unusual presentations of xanthomatosis in two patients with ALGS.
- This is the first case report demonstrating airway xanthomatosis in ALGS.
- Improvements in severe xanthomas and cholestatic pruritus were observed in patients with ALGS with maralixibat treatment.

## 1 INTRODUCTION

Alagille syndrome (ALGS) is a rare, autosomal dominant disease with hepatic manifestations, predominantly characterized by complications of cholestasis secondary to bile duct paucity. Key clinical manifestations of ALGS, which can lead to liver transplantation or death, include cholestasis, pruritus, failure to thrive, xanthomas, and progressive fibrosis.^1^ The presence of xanthomas is seen in 24%–42% of patients with ALGS.^1^, ^2^, ^3^, ^4^ Often a sign of a systemic disease, xanthomas are cutaneous lesions that result from an excessive uptake of low‐density lipoprotein (LDL) particles.^5^, ^6^ Xanthomas are thought to result from impaired bile acid excretion from the liver, which allows for the accumulation of cholesterol. Approximately 50% of patients with ALGS who receive a transplant identify xanthomas as an indication.^1^, ^2^, ^3^, ^4^ Ileal bile acid transporter (IBAT) inhibitors interrupt the enterohepatic circulation of bile acids, leading to increased bile acid excretion in feces. Maralixibat is approved for the treatment of cholestatic pruritus in children with ALGS aged 3 months and older in the United States and aged 2 months and older in the European Union.^7^, ^8^ Before this report, 14 participants in the ICONIC trial with xanthomas at baseline experienced significant improvements with maralixibat by Weeks 48 and 204.^4^ Herein, we provide a detailed account of two patients from two institutions, with ALGS complicated by cholestatic pruritus and xanthomas. Patients received maralixibat to treat their cholestatic pruritus either through a global Expanded Access Program, which facilitated access to maralixibat for patients with ALGS who could not participate in clinical trials, or through commercially available drug.

## 2 CASE REPORT

### 2.1 Case 1

**Figure 1:**
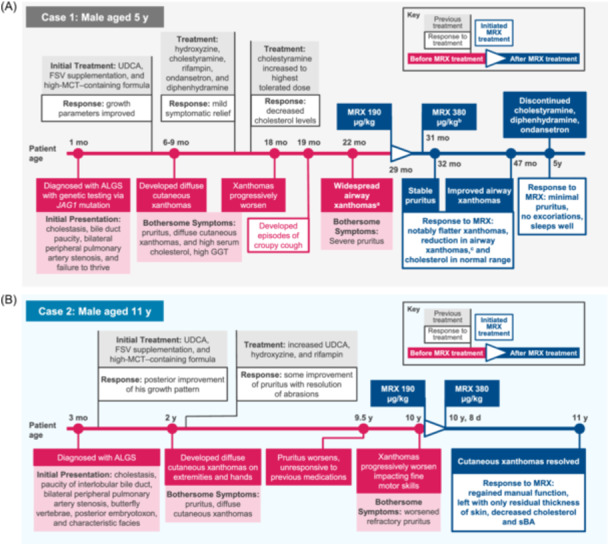
Timeline of patient clinical histories before and after maralixibat treatment. (A) 5‐year‐old male (patient case 1). (B) Eleven‐year‐old male (patient case 2). ^a^Seen with rigid bronchoscopy. ^b^After 1 week at 190 µg/kg maralixibat, dose was increased to 380 µg/kg. Dose was reduced to 190 µg/kg after 4 weeks due to persistent diarrhea. After 2 weeks at 190 µg/kg, diarrhea subsided, and the dose was again increased to 380 µg/kg. ^c^The patient was noted to have 4+ adenoids and 2–3+ tonsils and underwent adenoidectomy and tonsillectomy with improvement in cough noted postoperatively. ALGS, Alagille syndrome; FSV, fat‐soluble vitamin; GGT, gamma‐glutamyl transferase; MCT, medium chain triglyceride; MRX, maralixibat; sBA, serum bile acid; UDCA, ursodeoxycholic acid.

**Figure 2:**
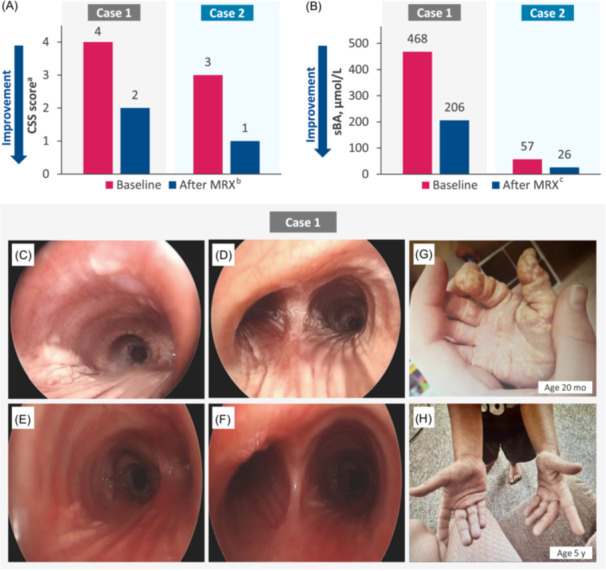
Clinical and laboratory parameters before and after maralixibat treatment. (A) CSS score in patients 1 and 2. ^a^CSS, 0 = none, 1 = rubbing or mild scratching when undistracted, 2 = active scratching without abrasions, 3 = abrasions, and 4 = cutaneous mutilations, hemorrhage, and scarring.^4^ ^b^At time of reporting, treatment duration for case 1 was 6 months and for case 2 was 12 months. (B) sBA levels in patients 1 and 2. ^c^At time of reporting, treatment duration was 12 months. (C) Rigid bronchoscopy in patient 1 at 22 months of age demonstrating widespread airway xanthomas in the distal trachea with extensive striping of the posterior tracheal wall with streaks of xanthomas and (D) extending into both mainstems at the carina. (E) Repeat bronchoscopy at 18 months after initiation of maralixibat treatment demonstrating notable improvement in airway xanthomas with minimal streaking in the left and right mainstem and (F) completely patent subglottis, no vocal cord lesions, and an almost clear trachea. (G) Xanthomas in patient 1 before (20 months of age) and (H) after (5 years old) maralixibat treatment. CSS, clinician scratch scale; MRX, maralixibat; sBA, serum bile acid.

Patient 1, a 5‐year‐old male, was diagnosed with ALGS at 1 month of age based on biochemical findings of cholestasis, histopathological findings of bile duct paucity, bilateral peripheral pulmonary artery stenosis, and presence of a *JAG1* variant (Figure 1A). He was started on ursodeoxycholic acid (UDCA), fat‐soluble vitamin supplementation, and high‐medium chain triglyceride (MCT)–containing formula for failure to thrive. At 6 months of age, he had a gastrostomy tube placed to facilitate growth. At 6–9 months of age, the patient developed severe pruritus with clinician scratch scale (CSS) score of 4, indicating cutaneous mutilation and diffuse cutaneous xanthomas. He was started on hydroxyzine, cholestyramine, and rifampin for his pruritus but only had mild symptomatic relief. Eventually, ondansetron and diphenhydramine were also added to his regimen for pruritus. At approximately 18 months of age, his xanthomas worsened, and he developed a recurrent croupy cough with subsequent rigid bronchoscopy demonstrating widespread airway xanthomas. Baseline assessments before treatment with maralixibat were: CSS score of 4; total cholesterol (TC), 404 mg/dL; total bilirubin (TB), 4.4 mg/dL; serum bile acids (sBA), 468 µmol/L; alanine aminotransferase (ALT), 111 U/L; and aspartate aminotransferase (AST), 103 U/L (Table 1). At baseline, LDL cholesterol (LDL‐C) was 325 mg/dL and conjugated bilirubin (CB) was 1.2 mg/dL. At 2.5 years of age, the patient received maralixibat 190 μg/kg/day for severe pruritus. The dose of maralixibat was increased to 380 μg/kg/day after 1 week. After 4 weeks of treatment, he developed diarrhea and some vomiting with negative infectious stool studies, and therefore, the maralixibat dose was decreased back to 190 μg/kg/day. Diarrhea and vomiting resolved with the lower dose, and maralixibat was then increased back to 380 μg/kg/day after 2 weeks. The patient did not develop recurrent gastrointestinal symptoms. After 6 months of treatment with maralixibat, the patient experienced clinically meaningful improvements in both pruritus (CSS: 2 [Δ: −2]) and sBA (206 µmol/L [Δ: −262 µmol/L]) (Figure 2A,B). After 18 months of treatment with maralixibat, he had a repeat bronchoscopy with significant improvements in airway xanthomas (Figure 2C–F). Specifically, xanthomas were observed to be significantly flatter. Additionally, improvements in levels of TC (389 mg/dL [Δ: −15]) and LDL‐C (248 mg/dL [Δ: −77]) were observed. Currently, after 2.5 years of treatment, the patient's xanthomas are resolved (Figure 2G,H), and pruritus is controlled with maralixibat daily, UDCA twice daily, rifampin twice daily, and hydroxyzine as needed, with a CSS score of 0 (Δ: −2). Trials removing or reducing rifampin utilization have been unsuccessful. However, removal of cholestyramine, diphenhydramine, and ondansetron was successful due to maralixibat's effectiveness in ameliorating the pruritus. He continues to have normal synthetic liver function. To our knowledge, this is the first case presented in the literature of upper and lower airway xanthomas in a child with ALGS secondary to hypercholesterolemia, which were markedly improved after initiation of maralixibat.

**Table 1:** Laboratory assessment values.

Laboratory assessment	Case 1		Case 2	
	Baseline	After MRX (Duration)	Baseline	After MRX (12 months)
CSS score^a^	4	2 (6 months)	3	1
sBA, µmol/L	468	206 (6 months)	57	26
ALT, U/L	111	90 (8 months)	103	58
AST, U/L	103	116 (8 months)	111	64
Total cholesterol, mg/dL	404	389 (11 months)	525^b^	271
Total bilirubin, mg/dL	4.4	5.3	0.3	0.1

Abbreviations: ALT, alanine aminotransferase; AST, aspartate aminotransferase; CSS, clinician scratch scale; MRX, maralixibat; sBA, serum bile acids.

^a^ CSS scores (0–4); 0 = none, 1 = rubbing or mild scratching when undistracted, 2 = active scratching without abrasions, 3 = abrasions, and 4 = cutaneous mutilations, hemorrhage, and scarring.^4^

^b^ Baseline assessment for case 2 was at 10 years of age.

### 2.2 Case 2

Patient 2, an 11‐year‐old male, was diagnosed with ALGS at 3 months of age based on biochemical findings of cholestasis and findings of paucity of interlobular bile ducts, bilateral peripheral pulmonary artery stenosis, and butterfly vertebrae (Figure 1B). He also had posterior embryotoxon and classical facial features of ALGS. His genetic testing did not reveal any variants in the *JAG1* or *NOTCH2* genes. He was started on UDCA, fat‐soluble vitamin supplementation, and high‐MCT–containing formula for failure to thrive, with posterior improvement of his growth pattern. By 2 years of age, he developed pruritus, diffuse cutaneous xanthomas on his extremities, particularly his hands, and hypercholesterolemia. His UDCA dose was increased, and he was started on hydroxyzine and rifampin for his pruritus, which provided limited improvement. By 9.5 years of age, his pruritus worsened, and he was unresponsive to his previous medications. At 10 years of age, before starting maralixibat, his cutaneous xanthomas started to worsen, particularly on his hands, impacting his fine motor skills. Baseline assessments before treatment with maralixibat were: CSS score of 3; TC, 525 mg/dL; TB, 0.3 mg/dL; sBA, 57 µmol/L; ALT, 103 U/L; and AST, 111 U/L (Table 1). He was started on maralixibat at 190 μg/kg/day and increased to 380 μg/kg/day by Day 8. After 12 months of treatment with maralixibat, he regained manual function, and his cutaneous xanthomas resolved, only leaving him with thicker skin on his hands. Patient 2 experienced clinically meaningful improvements in both pruritus (CSS: 1 [Δ: −2]) and sBA (26 µmol/L [Δ: −31 µmol/L]) (Figure 2A,B). Additionally, he demonstrated marked improvement in levels of TC (271 mg/dL [Δ: −254 mg/dL]). This case demonstrated the resolution of severe cutaneous xanthomas 12 months after initiation of maralixibat treatment, resulting in regained manual function.

## 3 DISCUSSION

Cholestatic liver disease in ALGS is characterized by several clinical features, including intrahepatic bile duct paucity, severe pruritus, and xanthomas.^3^, ^9^ Pruritus and xanthomas are considered some of the most bothersome symptoms of cholestatic liver disease in patients with ALGS. Xanthomas have been found to be associated with poor long‐term hepatic outcomes. In patients with a native liver, the presence of xanthomas is linked to a lower 10‐year survival rate than in those without.^1^, ^3^, ^10^ This is the first case report demonstrating the effectiveness and safety of an IBAT inhibitor in patients with ALGS with pruritus and severe, debilitating xanthomas. The ICONIC study by Gonzales et al. showed significant improvements in xanthomas in participants treated with maralixibat by 48 weeks, based on overall reductions in the Clinician Xanthomas Score, with further improvements in patients treated for longer durations. Xanthoma reduction was associated with improved quality of life and levels of TC.^4^ This report reviewed real‐world experience in two patients with ALGS and characterized the extent of their improvement after treatment with maralixibat. Specifically, one patient had improvement in tracheal xanthomas after maralixibat treatment, whereas the other patient had loss of function secondary to xanthomas that completely resolved after treatment.

## 4 CONCLUSIONS

In this case report, we describe the use of maralixibat in two patients with ALGS and unusual manifestations of xanthomatosis. The results reported herein suggest maralixibat may be a useful treatment option for patients with ALGS who have both pruritus and severe debilitating xanthomas.

## CONFLICT OF INTEREST STATEMENT

Ruben E. Quiros‐Tejeira is a consultant for Mirum Pharmaceuticals, Inc. Bernadette Vitola is a consultant for Mirum Pharmaceuticals, Inc. The remaining author declares no conflict of interest.

## ETHICS STATEMENT

Informed patient consent from the patients' guardians was obtained for publication of the case details.
